# Medication-focused telehealth interventions to reduce the hospital readmission rate: a systematic review

**DOI:** 10.1080/20523211.2025.2457411

**Published:** 2025-02-05

**Authors:** Fatemeh Emadi, Racha Dabliz, Rebekah Moles, Stephen Carter, Jenny Chen, Charu Grover, Manya Angley, Rohan A. Elliott, Deirdre Criddle, Deborah Rigby, Sepehr Shakib, Frank Sanfilippo, Charley Budgeon, Kim-Huong Nguyen, Paul Yates, Katie Phillips, Anna Packer, Linda Krogh, Simon Poon, Jonathan Penm

**Affiliations:** a Faculty of Medicine and Health, School of Pharmacy, The University of Sydney, Camperdown, Australia; b Australian Commission on Safety and Quality in Health Care, Sydney, Australia; c Charles Perkins Centre, The University of Sydney, Camperdown, Australia; d Manya Angley Research and Consulting; e Pharmacy Department, Austin Health, Melbourne, Australia; f Faculty of Pharmacy and Pharmaceutical Sciences, Monash University, Melbourne, Australia; g Pharmacy Department, Sir Charles Gairdner Hospital, Perth, Australia; h Complex Needs Coordination Team, South Metropolitan Health Service, Perth, Australia; i School of Pharmacy, University of Queensland, Woolloongabba, Australia; j School of Biomedicine, Faculty of Health Sciences, The University of Adelaide, Adelaide, Australia; k School of Population and Global Health, University of Western Australia, Perth, Australia; l Centre for Applied Health Economics, School of Medicine and Griffith Health Institute, Griffith University, Gold Coast, Australia; m Department of Molecular Imaging and Therapy, Austin Health, Heidelberg, Australia; n The Royal Melbourne Hospital, Hospital Admission Risk Program and cohealth Community Health Organisation, Melbourne, Australia; o Western NSW Local Health District, Dubbo, Australia; p Western NSW Local Health District, Orange, Australia; q School of Computer Science, The University of Sydney, Camperdown, Australia; r Department of Pharmacy, Prince of Wales Hospital, Randwick, Australia

**Keywords:** Telehealth, medication therapy, hospital readmission, transition of care

## Abstract

**Background:**

Transition of care from hospital to community is a period that carries significant risk for medication errors, potentially leading to hospital readmission, and causing financial and emotional strain on patients and caregivers. Telehealth technologies offer promising solutions to reduce hospital readmission. Therefore, the goal of this systematic review was to examine the effect of interdisciplinary telehealth post-discharge services that include a medication-focused component on hospital readmissions.

**Methods:**

Following the PRISMA guidelines, a search was conducted in five scientific databases using keywords related to hospital readmission, medication therapy, and telehealth interventions. The review focused on randomised controlled trials published between 2000 and 2023, written in English.

**Results:**

Out of 1,144 papers screened, 23 were included in the review. These studies targeted telehealth service provision to people over 60 years old with chronic illnesses. Various post-discharge telehealth interventions, including medication-focused components, were examined. Most of the interventions were multifaceted, embedded medication-focused interventions with reminders for diet, exercise, symptom check-ups, and education. Among the 23 included papers, 10 studies demonstrated success in reducing readmissions. Seven of these studies targeted patients with heart failure (HF).

**Conclusion:**

Overall, this review highlights the potential of telehealth medication-focused interventions in reducing hospital readmission rates in patients with HF.

## 1. Background

Transition of care is a key focus area in the World Health Organisation's (WHO) third global patient safety challenge, which aims to reduce medication-related harm (Sheikh et al., 2017). Transitioning from the hospital to community settings is particularly critical and poses a high risk of medication errors. A systematic review revealed that, on average, 53% of patients experience medication errors and 50% encounter unintentional medication discrepancies following discharge (Alqenae et al., 2020). These medication errors during post-discharge care transition significantly increase the likelihood of hospital readmission. According to Morabet's systematic review, the median rate of medication-related hospital readmissions was 21%, with 69% of them preventable (El Morabet et al., 2018). Several factors may contribute to the risk of medication-related hospital readmissions, including polypharmacy, medication non-adherence (Linkens et al., 2020) and taking high-risk medicines (Criddle et al., 2022). Polypharmacy increases the risk of drug interactions, adverse events, and low adherence due to complex regimens, leading to poor outcomes and higher readmission rates (Linkens et al., 2020). Medication non-adherence, whether intentional or unintentional, further exacerbates risks by contributing to disease progression or complications (Linkens et al., 2020). Moreover, the use of high-risk medicines presents additional challenges due to their narrow therapeutic index and significant potential for adverse effects frequently necessitating hospital-level care (Criddle et al., 2022). Hospital readmissions also strain the healthcare system financially and cause emotional distress for patients and families (Alzahrani, 2021; Axon & Williams, 2011). Effective post-discharge strategies are needed to improve medication safety and reduce readmissions.

Interventions to address medication-related readmissions include educational initiatives, clear communication of medication plans, and timely medication reviews (Linkens et al., 2020). Promoting communication among healthcare teams and between patients and caregivers has also been beneficial. Involving pharmacists, implementing patient educational programmes, and utilising transition-of-care interventions, along with the aid of digital tools like Clinical Decision Support Systems, are key components of these interventions (Becker et al., 2021). However, access and uptake challenges persist, particularly in remote areas with healthcare workforce shortages. These areas face inadequate handovers, insufficient monitoring, and limited resources, increasing the risk of medication errors (Calciolari et al., 2016; Ridge et al., 2022; Wang et al., 2022).

Telehealth technologies offer a promising solution to these challenges by providing patient education and improving access to care (Kuwabara et al., 2020). Telehealth is particularly valuable in remote areas with limited healthcare access, as it reduces costs, minimises travel time, and improves treatment outcomes (Cummings et al., 2019). For instance, telehealth enhances diabetes management by enabling timely treatment adjustments, promoting self-management, and improving cardiometabolic outcomes (Lee et al., 2023). In cardiovascular care, it supports improvements in physical activity, health behaviours, and medication adherence(Shi et al., 2024). Similarly, in mental health, telehealth facilitates access to counselling, cognitive behavioural therapy, and psychiatric care, while improving treatment adherence and reducing stigma (Lawes-Wickwar et al., 2018). Furthermore, a systematic review by Mashhadi et al. confirmed that mobile health interventions post-discharge positively impact reducing hospital readmissions (Mashhadi et al., 2021).

To our knowledge, there is no systematic review on the effect of telehealth post-discharge services that include a medication-focused component on hospital readmissions. So, this systematic review aims to evaluate the effectiveness of interdisciplinary multicomponent telehealth post-discharge services that include a medication-focused component on hospital readmissions.

## 2. Methods

This review followed the Preferred Reporting Items for Systematic Reviews and Meta-Analyses (PRISMA) guidelines for systematic reviews and meta-analyses (Moher et al., 2015). The protocol for the review was submitted for registration in PROSPERO (receipt number: 412674).

### 2.1. Research question

This systematic review was conducted to address the research question formulated using the Population, Intervention, Comparison, and Outcome (PICO) framework: ‘How effective were telehealth services, including medication-focused component, in reducing readmission rates to hospitals?’ Specifically, the review aimed to evaluate the impact of medication-focused telehealth interventions (Intervention) on reducing readmission rates (Outcome) among patients who had been discharged from various types of hospitals (Population). The review compared the telehealth services to usual care as a comparison (Comparison).

The interventions under investigation were designed to support patients following discharge from the hospital. These interventions may have included components such as medication review, sending reminders, medication adherence support, education and information, and virtual consultations delivered through telehealth. The interventions could be provided by healthcare professionals, such as pharmacists, medical practitioners, and nurses who were offering guidance and support that was focused on the optimal use of medications and ensuring implementation of the medication management plan.

### 2.2. Search strategy

The search involved five databases: PubMed, Scopus, ProQuest, Web of Science, and Embase. These databases were selected for their comprehensive coverage of biomedical, clinical, and multidisciplinary research, ensuring a wide scope of relevant and high-quality studies. English journal papers published between January 1, 2000, and March 14, 2023, involving patients who had been discharged from any type of hospital. The focus was on post-discharge telehealth interventions with medication-focused components aimed at reducing hospital readmission rates. Randomised Controlled Trials (RCTs) were included as the eligible study designs because they provide the highest level of evidence in evidence hierarchies. Lower-level evidence, such as that from cross-sectional studies, conference abstracts, and opinion articles or editorials, was excluded to maintain the reliability of the findings (Burns et al., 2011). Supplemental Material S1 contains the comprehensive and detailed search strategies employed for each of the databases. The main outcome of interest was the reduction in hospital readmission rates.

### 2.3. Data extraction

The Covidence platform (https://www.covidence.org) was utilised for managing the systematic review process. Following the removal of duplicate articles, two authors independently reviewed the titles and abstracts of the remaining articles using the Covidence platform to identify potentially suitable studies. The full-text articles were subsequently evaluated within Covidence to determine their eligibility. In the event of any disagreements, a third author was consulted to reach a consensus. The process of data extraction followed the PRISMA standard form (Moher et al., 2015). The extracted information included details such as author names, publication year, country, time of service delivery, type of hospital, study size, participants’ characteristics (including intervention and control groups), type of intervention and medication-focused services, control services, related healthcare professionals involved, age of participants, disease studied, baseline status, single or multi-centre study, design of the RCT, length of follow-up, randomisation generation and concealment methods, outcome status and measure of readmission, results related to hospital readmission outcomes, other relevant outcomes, funding sources, comments on missing or unclear information, and registration status.

### 2.4. Quality assessment

Two authors independently assessed the quality of all included studies. The Cochrane Risk of Bias Assessment Tool (RoB 2) (Sterne et al., 2019) was used as our study included only randomised controlled trials, for which this tool is specifically designed to assess methodological quality. The studies were categorised into different levels of bias risk (low, high and some concerns) based on five domains including randomisation process, deviation from intended interventions, missing outcome data, measurement of the outcome and selection of the reported result.

### 2.5. Data synthesis

The complexity of each multidisciplinary intervention underwent assessment by two authors using the Cochrane Intervention Complexity Assessment Tool for Systematic Reviews (iCAT_SR) (Lewin et al., 2017). The authors assigned scores of 0–3 to various criteria in each of the six domains, with a score of less than 6 indicating the lowest level of complexity, 6–11 medium level of complexity and 12 and above representing the highest level of complexity. The criteria encompassed factors such as the number of intervention components, targeted recipient behaviour, organisational levels targeted, required tailoring, and the level of skill needed for intervention delivery and receipt. The studies were categorised based on the type of medication-focused interventions and their effectiveness in reducing hospital readmission rates for specific diseases. Heterogeneity among the studies was evaluated through the comparison of the intervention approach, and hospital readmission measurement method. Due to variations in interventions and reported outcomes, the researchers were unable to conduct a meta-analysis.

## 3. Results

**Figure 1.**
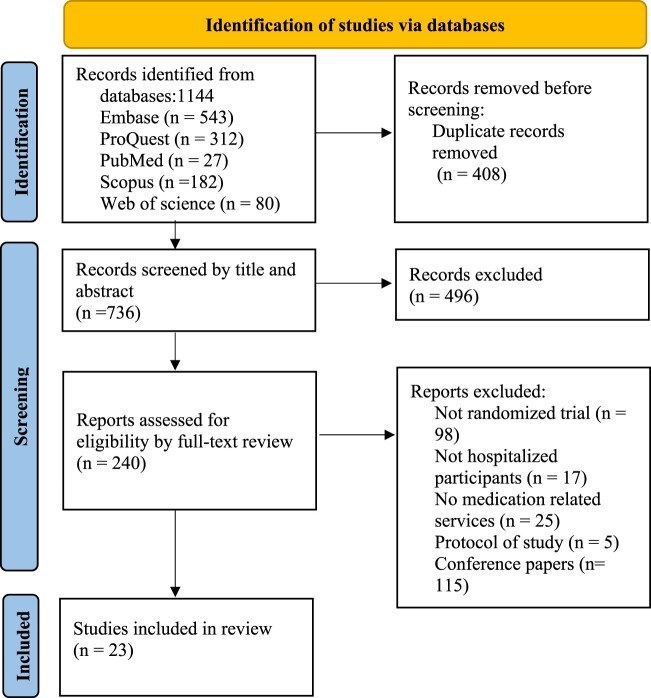
PRISMA flow chart of the study selection.

The initial database search yielded a total of 1,144 articles. After removing 408 duplicates, 736 papers were screened based on their titles and abstracts (Figure 1). Following this screening, 240 studies were considered eligible for full-text review. Among them, 217 papers were excluded due to factors such as non-RCT design, non-hospitalized patients, protocol of study, conference papers and absence of medication-focused services. Ultimately, 23 studies met the systematic review criteria. Risk of bias assessment using the Rob2 tool was conducted for all 23 RCTs (Table 1). Seven studies had a high risk of bias due to issues such as missing data, inconsistent baseline of control and treatment group and self-reported data while 16 papers raised some concerns (Supplemental Material S2).

**Table 1.** Risk of bias assessment of studies using ROB2.

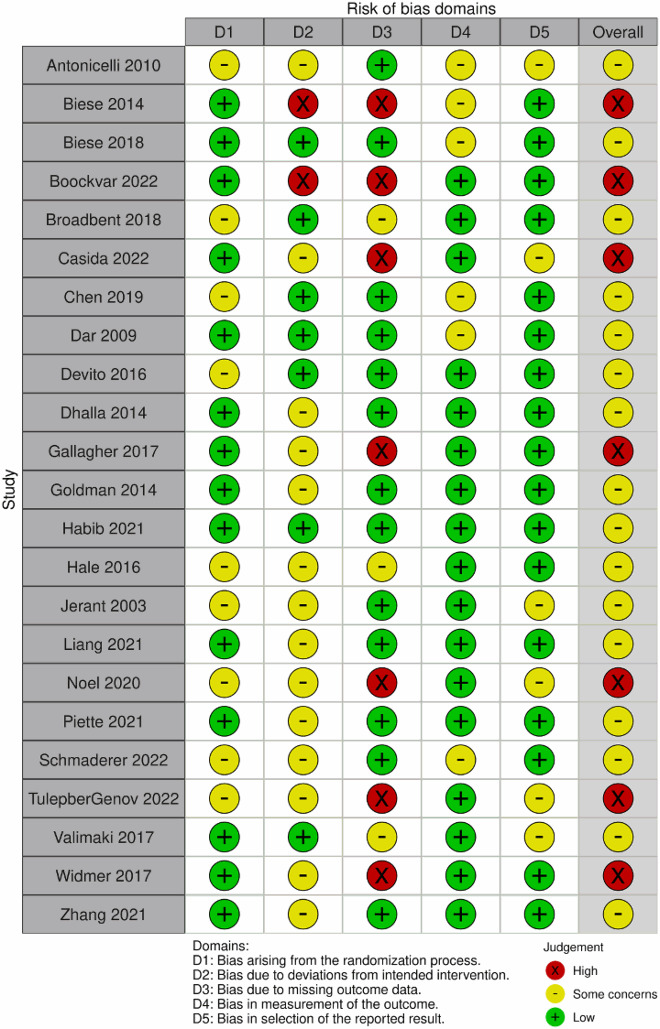

Table 2 shows the findings of the systematic review. Several included studies focused on chronic diseases, such as Heart Failure (HF) (*n* = 8) (Antonicelli et al., 2010; Chen et al., 2019; Dar et al., 2009; Gallagher et al., 2017; Hale et al., 2016; Jerant et al., 2003; Schmaderer et al., 2022; Tulepbergenov et al., 2022), Chronic Obstructive Pulmonary Disease (COPD) (Broadbent et al., 2018), Lung Transplants (DeVito Dabbs et al., 2016), cancer (Zhang et al., 2021), and multiple chronic illnesses (Liang et al., 2021) and 11 studies did not specify the types of diseases (Biese et al., 2014, 2018; Boockvar et al., 2022; Casida et al., 2022; Dhalla et al., 2014; Goldman et al., 2014; Habib et al., 2021; Noel et al., 2020; Piette et al., 2021; Välimäki et al., 2017; Widmer et al., 2017). More than half of the studies (13 out of 23) were conducted in the USA (Biese et al., 2014, 2018; Boockvar et al., 2022; Casida et al., 2022; DeVito Dabbs et al., 2016; Gallagher et al., 2017; Goldman et al., 2014; Hale et al., 2016; Jerant et al., 2003; Noel et al., 2020; Piette et al., 2021; Schmaderer et al., 2022; Widmer et al., 2017).

**Table 2.** Findings extracted from the included studies in the systematic review.

Author, Year, Country	Study size/ Disease	Telehealth method	Medication Services		Outcome Measure (Day/ Month)	Hospital readmission Results	Non-medication services	Frequency
			Adherence	Management				
Antonicelli et al., 2010, Italy	57 (I:29, C:28) / HF	Phone call	Yes	Yes	12m	*I* = 31%, C = 93%, *p* = 0.05	Vital sign monitoring, ECG monitoring, Symptoms monitoring	Weekly
Biese et al., 2014, USA	120 (I:39, C:46, Placebo-35)/ NI	Phone call	Yes	Yes	35d	*I* = 22%, C = 27%, placebo = 33%, *p* = 0.41	Post-discharge review, Follow-up reinforcement, Health status advice	Once, Day1-3; post-discharge
Biese et al., 2018, USA	1949 (I:974, C:975)/ NI	Phone call	Yes	Yes	30d	*I* = 9% (95% CI = 7.2-10.8%), C = 7.4% (95% CI = 5.8-9.0%)	Post-discharge review, Follow-up reinforcement, Health status advice	Once, Day1-3 post-discharge
Boockvar et al., 2022, USA	347 (I:159 C:188)/ NI	Phone call	Yes	No	90d	*I* = 25.8%, C = 20.2%; Risk Difference 5.6% (95% CI = 3.3-14.5%), *p* = 0.25	Education on condition, Symptoms monitoring	Monthly
Broadbent et al., 2018, NZ	60 (I:30, C:30)/ COPD	Telemonitoring equipment	Yes	No	120d	*I* = 50%, C = 50%, *p* > 0.99	Vital sign monitoring, Symptoms monitoring, Exercise reminder, Education on condition	Daily
Casida et al., 2022, USA	30 (I:14, C:16)/ NI	Mobile app	Yes	Yes	180d	*I* = 0.3% ± 0.61, C = 0.9% ± 0.93, *p* = 0.029	Education on condition, Vital sign monitoring, Symptoms monitoring, Dietary and fluid intake monitoring, Blood test monitoring	Daily
Chen et al., 2019, China	767 (I-SMS: 252, I-STS: 255, C:260)/ HF	SMS, Phone call	Yes	No	180d	SMS = 33.7%, Phonecall = 30.6%, C = 42.7%. Odds Ratio: SMS vs C-0.790 (95% CI = 0.632, 0.988), *p* = 0.037; Phonecall vs C-0.716 (95% CI = 0.568, 0.904), *p* = 0.004; SMS vs Phonecall-1.103 (95% CI = 0.856, 1.421)	Education on condition, Lifestyle education, Follow-up reinforcement	Day10 post-discharge, weekly for 1 month, then follow up in 180days (not regularly)
Dar et al., 2009, UK	182 (I:91, C:91)/ HF	Telemonitoring equipment	Yes	Yes	180d	*I* = 36%, C = 25%, Proportion of emergency heart failure hospitalization: *I* = 36%, C = 81%, *p* = 0.01 / Duration of hospitalization (median, IQR): *I* = 17 (6-25), C = 13 (8-34), *p* = 0.99	Vital sign monitoring, Lifestyle education	Daily
DeVito Dabbs et al., 2016, USA	201 (I:99 C:102)/ Lung Transplant	Mobile app	Yes	No	12m	*I* = 81%, C = 85%, *p* = 0.51	Symptoms monitoring, feedback messages	Daily
Dhalla et al., 2014, Canada	1932 (I:967 C:965)/ NI	Phone call	No	Yes	12m	*I* = 18.9%, C = 21.3%, *p* = 0.21	Post-discharge interventions delivered as virtual ward	Daily up to discharge from virtual ward
Gallagher et al., 2017, USA	40 (I:20, C:20)/ HF	Phone call	Yes	No	30d	*I* = 30%, C = 20%, *p* = 0.72	Not Applicable	Daily for one week and weekly for 3 weeks
Goldman et al., 2014, USA	700 (I:353, C:347)/ NI	Phone call	Yes	Yes	30d	30-day HRA Hazard Ratio = 1.17 (95% CI = 0.79-1.74), *p* = 0.43; 90-day HRA Hazard Ratio = 1.11 (95% CI = 0.79-1.56), *p* = 0.54; 180-day HRA Hazard Ratio = 0.97 95% (95% CI = 0.73-1.29), *p* = 0.83	Education on condition, Follow-up reinforcement, post-discharge review	Day 1, 3, 6, 10, 30 post-discharges
Habib et al., 2021, Canada	49 (I:23, C:26)/ NI	Mobile app	Yes	Yes	30d	*I* = 8.7%, C = 15.4% *P* < 0.05	Not Applicable	Daily
Hale et al., 2016, USA	29 (I:13, C:16)/HF	Telemonitoring equipment and Phone call	Yes	Yes	90d	*I* = 50%, C = 9%, *p* = 0.04	Not Applicable	Daily telemonitoring and phone call as needed
Jerant et al., 2003, USA	37 (I-1:13, I-2:12, C:12)/ HF	Telemonitoring equipment and Phone call	Yes	Yes	180d	HF-related readmission charges were > 80% lower in the telenursing groups compared to usual care, and these groups also had significantly fewer HF-related emergency visits.	Vital sign monitoring, Dietary and exercise monitoring	About 60 days out of 180days (order not mentioned)
Liang et al., 2021, Taiwan	200 (I:100, C:100)/ Chronic illness	Telemonitoring equipment, mobile app	Yes	No	180d	*I* = 44%, C = 41%, Odds Ratio = 1.131 (95% CI = 0.645-1.981), *p* = 0.668	Vital sign monitoring, Emergency assistance service	Twice daily
Noel et al., 2020, USA	74 (I:31, C:43)/ NI	Telemonitoring equipment, mobile app	Yes	Yes	12m	Regression estimate = 2.645 (95% CI = 0.404-17.328), *p* = 0.31	Vital sign monitoring	Weekly video visits with daily remote patient monitoring
Piette et al., 2021, USA	284 (I:141, C:143)/ NI	Automated Interactive Voice Response	Yes	No	90d	*I* = 20.8%, C = 32.2% readmission, *p* = 0.41	Education on condition, Follow-up reinforcement, exercise follow up	Daily (2 weeks post discharge) 3 times weekly (second 2 weeks) and then once weekly(for 9 weeks)
Schmaderer et al., 2022, USA	74 (I-1: 26, I-2:27, C:27)/ HF	Mobile app with virtual visits	Yes	No	90d	Fisher exact test statistic = 0.017, *p* = 0.02	Education on condition, Lifestyle education, Symptoms monitoring, Follow-up reinforcement	Weekly video visits with daily patient monitoring
Tulepbergenov et al., 2022, Kazakhstan	599 (I:322, C:277)/HF	Mobile app	Yes	No	12m	*I* = 9.8%, C = 23.1% readmission, Relative Risk = 0.41 (95% CI = 0.19-0.59), *p* = 0.024	Not Applicable	Daily
Välimäki et al.,^2017^, Finland	1139 (I:569, C:570)/ NI	SMS	Yes	No	12m	*I* = 43.0%, C = 38.8%; Relative Risk: 1.11 (95% CI: 0.92-1.33), *p* = 0.28	Appointment reminder Self-care reminder	12 per month
Widmer et al., 2017, USA	71 (I:37, C:34)/ NI	Mobile app	Yes	No	180d	*I* = 8.1%, C = 26.6%; Relative Risk = 0.30 (95% CI = 0.08-1.10), *p* = 0.054	Lifestyle education, Vital sign monitoring, Blood test monitoring	Daily
Zhang et al., 2021, China	100 (I:49, C:51)/ Cancer	Mobile app	Yes	Yes	30d	χ2 = 0.010; *P* = 0.92	Not applicable	Daily

HF: heart failure, COPD: chronic obstructive pulmonary disease, NI: disease not specified, ED: emergency department, SMS: short message service, STS: structured telephone support, d: day, m: month, HRA: hospital readmission, I: intervention, C: control, CI: confidence interval, Medication management: activities such as dose adjustment, medication advice, remote visits with pharmacists, medication reconciliation and remote dispensing or procurement of medication

The RCTs included in the review employed various medication-focused telehealth interventions (Table 2). These were delivered through telemonitoring equipment (Broadbent et al., 2018; Dar et al., 2009; Hale et al., 2016), phone calls (Antonicelli et al., 2010; Biese et al., 2014, 2018; Boockvar et al., 2022; Dhalla et al., 2014; Gallagher et al., 2017), short text messages (SMS) (Chen et al., 2019; Välimäki et al., 2017), mobile apps (Casida et al., 2022; DeVito Dabbs et al., 2016; Habib et al., 2021; Tulepbergenov et al., 2022; Widmer et al., 2017; Zhang et al., 2021), and Automated Interactive Voice Response (IVR) systems (Piette et al., 2021) or combination of these (Jerant et al., 2003; Lawes-Wickwar et al., 2018; Liang et al., 2021; Noel et al., 2020; Schmaderer et al., 2022).

Telemonitoring equipment, including devices such as scales, blood pressure cuffs, pulse oximeters, and medication monitoring systems, enabled patients to monitor their vital signs and medication adherence from the comfort of their homes. Data, including weight, blood pressure, heart rate, oxygen saturation, and medication usage, was securely transmitted to healthcare providers via dedicated lines or Wi-Fi. Alerts were triggered if measurements fell outside normal ranges or if medication doses were missed, prompting timely interventions. Additionally, these systems provided educational resources on health conditions and medication information, reminders for medication and exercise, and facilitated direct communication with healthcare professionals. This integrated approach enhanced patient adherence, supported early detection of potential health issues, and ensured that treatment plans could be promptly adjusted based on real-time data (Broadbent et al., 2018; Dar et al., 2009; Hale et al., 2016).

The medication-focused interventions mainly addressed medication adherence in 11 studies (Boockvar et al., 2022; Broadbent et al., 2018; Chen et al., 2019; DeVito Dabbs et al., 2016; Gallagher et al., 2017; Liang et al., 2021; Piette et al., 2021; Schmaderer et al., 2022; Tulepbergenov et al., 2022; Välimäki et al., 2017; Widmer et al., 2017) medication management only in one study (Dhalla et al., 2014) or a combination of both medication adherence and management in 11 studies (Biese et al., 2014, 2018; Casida et al., 2022; Dar et al., 2009; Goldman et al., 2014; Habib et al., 2021; Hale et al., 2016; Jerant et al., 2003; Noel et al., 2020; Zhang et al., 2021). The interventions targeting medication adherence specifically aimed to support or monitor adherence to prescribed medication dosages or regimens by tracking the adherence, sending reminders or discussing the reasons of non-adherence. On the other hand, the medication management interventions included activities such as dose adjustment, medication advice, remote visits with pharmacists, medication reconciliation and remote dispensing or procurement of medications. As mentioned earlier, some studies incorporated interventions that targeted both adherence support and medication management services.

Out of the 23 studies included, a majority (21 studies) employed multi-component interventions. No study demonstrated a low level of complexity, whereas nine of these studies displayed a high level of complexity, and the remaining 13 papers exhibited a medium level of complexity, as assessed by iCAT_SR (Supplemental Material S3). The details of multi-component studies are summarised in Table 2. Among 10 successful studies, five studies were highly complex (50%) while among the 13 unsuccessful studies, four studies were highly complex (30%) and the nine remaining studies showed medium level of complexity (Supplemental Material S3). These multi-component approaches encompassed a range of complementary telehealth services provided in conjunction with medication-focused services. These services included vital sign monitoring, ECG monitoring, symptoms monitoring, post-discharge review, follow-up reinforcement, health status advice, education on the patient's health condition, exercise reminders, dietary and fluid intake monitoring, blood test monitoring, lifestyle education, and emergency assistance services.

Regarding the frequency, daily multidisciplinary interventions were the most prevalent frequency that was employed in 10 studies (Broadbent et al., 2018; Casida et al., 2022; Dar et al., 2009; DeVito Dabbs et al., 2016; Dhalla et al., 2014; Habib et al., 2021; Hale et al., 2016; Tulepbergenov et al., 2022; Widmer et al., 2017; Zhang et al., 2021). Twice daily services also were employed in one study (Liang et al., 2021). Two studies used a combination of daily interventions for a fixed number of days followed by subsequent weekly interventions (Gallagher et al., 2017; Piette et al., 2021). While another two employed both daily intervention and weekly virtual visits simultaneously (Noel et al., 2020; Schmaderer et al., 2022). Furthermore, there were studies with less frequent interventions, such as every 2 days for 10 days followed by a single call on day 30 (Goldman et al., 2014), weekly (Antonicelli et al., 2010; Chen et al., 2019), monthly interventions (Boockvar et al., 2022), and interventions consisting of a single call only (Biese et al., 2014, 2018). Moreover, two studies did not mention a regular sequence of interventions, instead indicating occurrences of 12 per month (Välimäki et al., 2017) or every 60 days within a 180-day timeframe (Jerant et al., 2003).

All the included studies reported all-cause hospital readmission, and no study reported whether readmission was specifically medication-focused. The type of medication-focused telehealth interventions (e.g. phone call, mobile app, telemonitoring equipment) did not show any association with the outcomes. When analysing the reduction in hospital readmission rates, it was observed that 13 studies did not yield statistically significant results, while 10 studies showed a reduction in hospital readmission rates in the intervention groups. This outcome could potentially be linked to the quality of the studies. Almost 40% (5 out of 13) of the unsuccessful studies demonstrated a high risk of bias, while the remaining studies (8 out of 13) exhibited some concerns. Among the 10 successful studies, two studies (20%) showed a high risk of bias, and the rest had some concerns. This analysis suggests that the presence of high risk of bias may influence the outcomes of studies. The proportion of studies with high risk of bias is notably higher among those with non-significant results compared to those with significant results. However, it is worth noting that nine out of the 10 studies that had a positive impact on hospital readmission adopted more than one component intervention delivered as a bundle. This finding highlights the importance of taking a comprehensive approach to supporting post-discharge medication services.

Furthermore, among the 10 studies that showed a reduction in hospital readmission rates in the intervention groups, seven specifically targeted patients with HF. In 10 effective studies, four studies focused on providing medication adherence support alone(Chen et al., 2019; Piette et al., 2021; Schmaderer et al., 2022; Tulepbergenov et al., 2022); however, six of them (60%) combined it with broader medication management(Antonicelli et al., 2010; Casida et al., 2022; Dar et al., 2009; Habib et al., 2021; Hale et al., 2016; Jerant et al., 2003). Meanwhile, in 13 ineffective studies, five studies (38%) delivered a combination of medication management services with medication adherence support (Biese et al., 2014, 2018; Goldman et al., 2014; Noel et al., 2020; Zhang et al., 2021), and eight studies only provided medication adherence support (Boockvar et al., 2022; Broadbent et al., 2018; DeVito Dabbs et al., 2016; Dhalla et al., 2014; Gallagher et al., 2017; Liang et al., 2021; Välimäki et al., 2017; Widmer et al., 2017). Additionally, seven out of 10 effective studies measured hospital readmission rates over a timeframe of 3–6 months. Among the 12 studies that did not demonstrate a positive impact on hospital readmission, it was found that nine of them conducted follow-ups with patients for a duration of 30 days or 12 months. This suggests that a mid-term duration of interventions, coupled with medication adherence support and a muti-component approach, may be beneficial for patients.

## 4. Discussion

Post-discharge medication-focused telehealth service interventions alone are unlikely to achieve a significant reduction in hospital readmission rates. However, multifaceted interventions that included a medication-focused services have shown a higher likelihood of success in reducing readmissions. The results obtained in our systematic align with the findings of Kansagara et al.'s umbrella review and Morkisch et al.'s systematic review, which both suggest that the implementation of multi-component post-discharge services and high-intensity, multidisciplinary interventions effectively contribute to the reduction of readmission rates (Kansagara et al., 2015; Morkisch et al., 2020). However, none of the above reviews focused only on medication-focused telehealth interventions. Additionally, another study supports the idea that multi-component approaches, such as mobile health interventions and teach-back communication, have a positive and significant impact on hospital readmissions (Mashhadi et al., 2021). While this study primarily examined telehealth interventions and did not specifically emphasise on medication-focused interventions, its findings still provide support for the effectiveness of remote multifaceted post-discharge interventions in reducing all-cause hospital readmissions. Similarly, eight out of 10 included studies in this review that were effective at reducing all-cause readmission employed a holistic approach by combining medication interventions with non-medication services. Effective interventions were often complex and included medication-focused components (e.g. medication management, medication adherence support, and streamlined communication with healthcare provider) along with non-medication services (e.g. patient education emphasising self-care, symptom monitoring, lifestyle and medical advice). Therefore, a comprehensive approach that integrates both pharmacological and non-pharmacological interventions may reduce the all-cause hospital readmission rates. Given that none of the studies specifically reported medication-related hospital readmissions, it is recommended for future research to incorporate medication-related readmissions as a distinct subset within the wider scope of all-cause hospitalisation. This approach would enable an exploration into whether the inclusion of a medication-focused component in telehealth post-discharge services could contribute to the reduction of medication-related hospital readmissions.

Medication adherence support was identified as a component of all telehealth post-discharge multifaceted interventions. Adherence to medication regimens is a critical aspect of self-care, particularly among patients who have polypharmacy and are older in age. Poor medication adherence is a common issue in healthcare, and it can significantly impact treatment outcomes and patient overall health. Nonadherence to medications is associated with an increased risk of all-cause hospitalisation and mortality in older adults and a considerable financial burden on the healthcare system (Walsh et al., 2019). Rosen et al. evaluated medication adherence as a predictor of hospital readmission assessed by the Morisky medication adherence scale. The findings of this study indicate that patients with low and moderate medication adherence had a 2.54 times higher likelihood of readmission, after adjusting for other factors, compared to patients with high adherence (Rosen et al., 2017). A scoping review has shown telehealth interventions may be an effective strategy to improve medication adherence, particularly in older adults (Emadi et al., 2022). Across studies identified in our review that used telehealth strategies to reduce hospital readmission, the interventions specifically targeted medication adherence, either as a standalone intervention or in conjunction with medication management. Hence, the findings emphasise the significance of incorporating medication adherence into telehealth interventions aimed at reducing hospital readmissions.

The findings of this systematic review reveal that telehealth multi-component interventions that include medication-focused services are effective in reducing hospital readmissions for patients with HF. In our systematic review seven out of 10 studies that reduced hospital readmissions were conducted in HF patients, while in studies where no effect on readmissions was demonstrated, only one out of 13 studies focused on HF patients. Systematic review and meta-analysis studies on telehealth interventions for HF after hospital discharge, while not specifically focusing on medication-focused interventions, aligns with our findings by demonstrating a significant reduction in all-cause mortality, HF-related hospital admissions, length of hospital stays, and mortality in HF patients through home-based tele-monitoring (Masotta et al., 2024; Pandor et al., 2013; Zhu et al., 2020). Additionally, Umeh et al.'s systematic review demonstrated that telemonitoring reduces all-cause and heart failure-related hospitalisations (Umeh et al., 2022), and Lin et al.'s study also reported the effectiveness of telehealth interventions in HF management (Lin et al., 2017). Additionally, a systematic review of RCTs demonstrated that virtual ward transition systems, when compared to usual post-discharge services, are associated with a significant reduction in deaths and hospital readmissions among patients with HF (Chauhan & McAlister, 2022). None of above-mentioned systematic reviews specifically considered the medication component in their inclusion criteria, but their finding was aligned with our systematic review regarding the reduction in hospital readmission for HF patients. Managing heart failure often requires prescribing multiple medications, increasing the risk of polypharmacy with a prevalence ranging from 17.2% to 99% in individuals with HF (Beezer et al., 2022). Polypharmacy may explain why telehealth interventions focusing on medication adherence and management are particularly effective in these patients compared to treatments for conditions like COPD, which have fewer pharmacological options for disease modification. Moreover, non-adherence to the medications used for HF is more likely to result in acute deterioration (Wu & Moser, 2018). HF also associated with multimorbidity where the regular follow up, attention to medication management would also benefit other health conditions the patient has e.g. hypertension and renal dysfunction (Takeuchi et al., 2022). These interventions provide the ability to remotely monitor medication regimens, offer timely interventions when needed, and deliver personalised support. Overall, it seems that HF patients may benefit the most from multicomponent telehealth post-discharge services that include a medication-focused component in reducing hospital readmission. However, implementing of telehealth interventions faces multiple challenges, especially in remote and rural regions. These include limited internet access, a lack of awareness or understanding among consumers on how to use telehealth, restricted access to clinicians offering telehealth services, and inadequate resources on the patient's side (St Clair & Murtagh, 2019). Therefore, it is essential to address these obstacles when developing telehealth interventions for rural and remote areas.

This systematic review has several limitations that should be acknowledged. The included studies exhibited diversity in terms of patient populations, chronic diseases, interventions, and outcome measures, which prevented a meta-analysis and required a qualitative synthesis. Approximately 30% of the studies were found to have a high risk of bias, which could impact the validity of the findings due to issues such as missing data and unclear randomisation processes. The wide range of medication and non-medication interventions used in the studies posed challenges in determining the specific effects of individual interventions on hospital readmission rates. Additionally, the concentration of studies in the USA introduced a geographical bias, potentially limiting the generalizability of the findings to other healthcare systems and populations. Considering these limitations, it is evident that further research is needed to investigate the effectiveness of telehealth medication-focused interventions in reducing rehospitalization rates. It is important to acknowledge the complexities and inconsistencies encountered in the analysed studies. Therefore, a comprehensive understanding of the potential impact of telehealth medication-focused interventions on rehospitalization rates requires additional investigation that considers these multifaceted elements.

## 5. Conclusion

In conclusion, this systematic review highlights the potential of medication-focused telehealth interventions, as part of broader post-discharge strategies, to reduce hospital readmission rates, particularly in HF patients. These results have important implications for healthcare providers aiming to improve post-discharge outcomes, policymakers seeking to implement effective and scalable telehealth programmes, and patients who can benefit from enhanced care and reduced hospitalisations. However, the heterogeneity in patient populations, chronic conditions, interventions, and outcome measures across the studies prevented a meta-analysis. Future studies should focus on medication-related readmissions as an outcome, to further investigate and clarify whether including a medication component in telehealth post-discharge interventions effectively reduces medication-related readmissions. Additionally, there is a need for qualitative synthesis to explore in greater depth the mechanisms behind these interventions and to provide a clearer understanding of how they influence medication-related outcomes and hospital readmission rates.

## Supplementary Material

Supplemental Material S1

Supplemental Material S2

Supplemental Material S3
