# Supplementary material for: Medication-focused telehealth interventions to reduce the hospital readmission rate: a systematic review
Source: J Pharm Policy Pract. 2025 Feb 5;18(1):2457411. doi: 10.1080/20523211.2025.2457411 (PMC11800339; doi:10.1080/20523211.2025.2457411)
Supplement: Supplemental Material S1 [file JPPP_A_2457411_SM0364.docx]

| **Population** | **Intervention** | **Outcome** | **Context** |
| --- | --- | --- | --- |
| Hospital(s) | remote Interventions | Hospital readmission | Medication therapy |
|  | Digital Intervention(s) | Recurrent admission* | Medication* |
|  | Remote Intervention(s) | Readmission* | Medication therapy |
|  | Telecare tele-care | Return to hospital | Drug therapy |
|  | Telemonitoring tele-monitoring | Repeat admission* | Hospital pharmacy service* |
|  | Teletherapy tele-therapy | Frequent admission* | Clinical pharmacology |
|  | Telepharmacy tele-pharmacy | Second admission | Clinical pharmacy |
|  | Telemedicine | Patient readmission* | Drug information service* |
|  | Telenursing | Rehospitalization* | Medication therapy management |
|  | Virtual Medicine | Rehospitalisation* | Medication review |
|  | Mobile Health | Unplanned Hospital | Medication reconciliation |
|  | mHealth | Hospital Readmission* | Medication management |
|  | Telehealth | Readmission* | Medication system* |
|  | eHealth |  |  |
|  | Tele-nursing |  |  |
|  | Tele-medicine |  |  |
|  | Remote consultation |  |  |
|  | Distance counselling |  |  |
|  | Teleconsultation Tele-consultation |  |  |
|  | Electronic prescription* |  |  |
|  | Electronic prescribing |  |  |
|  | Electronic pharmacy |  |  |
|  | Remote pharmacy |  |  |
|  | Digital pharmacy |  |  |
|  | Digital health |  |  |
|  | Digital medicine |  |  |
|  | Digital nursing |  |  |
|  | e-medicine emedicine |  |  |
|  | Virtual nursing |  |  |
|  | Remote nursing |  |  |
|  | Virtual healthcare |  |  |
|  | Remote healthcare |  |  |

**Table A- Keywords**

**Table B- Embase search strategy and keywords**

|  | **Subheading** | **Keyword** |
| --- | --- | --- |
| **Population** | | |
| Hospital* |  | y |
| **Outcome** | | |
| Recurrent admission* |  | y |
| Readmission* |  | y |
| Return to hospital |  | y |
| Repeat admission* |  | y |
| Frequent admission* |  | y |
| Second admission |  | y |
| Patient readmission* |  | y |
| Rehospitalization* |  | y |
| Rehospitalisation* |  | y |
| Unplanned Hospital Readmission* |  | y |
| Hospital Readmission* | y | y |
| **Intervention** | | |
| Digital Intervention* |  | y |
| teleconsultation | y | y |
| Tele-consultation |  | y |
| Remote Intervention* |  | y |
| Telecare | y | y |
| Tele-care or tele care both same |  | y |
| Telemonitoring | y | y |
| Tele-monitoring, tele monitoring same |  | y |
| Teletherapy | y | y |
| Tele-therapy, tele therapy same |  | y |
| Telepharmacy | y | y |
| Tele-pharmacy |  | y |
| Telemedicine | y | y |
| telemedicine robot | y | y |
| Telenursing | y | y |
| tele-nursing |  | y |
| Tele medicine tele-medicine both same result |  | y |
| Virtual Medicine |  | y |
| Mobile Health |  | y |
| (Mobile health application) | y | y |
| mHealth |  | y |
| m-health |  | y |
| Telehealth | y | y |
| Tele health, tele-health |  | y |
| eHealth |  | y |
| e-health |  | y |
| Distance counselling |  | y |
| Remote consultation |  | y |
| Electronic prescribing | y | y |
| Electronic prescription* |  | y |
| Electronic pharmacy |  | y |
| Remote pharmacy |  | y |
| Digital pharmacy |  |  |
| Digital health |  | y |
| Digital medicine |  | y |
| Digital nursing |  | y |
| e-medicine |  | y |
| emedicine |  |  |
| Remote nursing |  | y |
| (Rural health nursing) | y | y |
| Virtual healthcare |  | y |
| Remote healthcare |  | y |
| **Context** | | |
| Medication* |  | y |
| Medication therapy |  | y |
| Drug therapy | y | y |
| Hospital pharmacy service* |  | y |
| Clinical pharmacology | y | y |
| Clinical pharmacy | y | y |
| Drug information service* |  | y |
| Medication therapy management | y | y |
| Medication review |  | y |
| Drug utilization review | y | y |
| Drug utilisation review |  | y |
| Medication reconciliation |  | y |
| Medication management |  | y |
| Medication system* |  | y |

**Table C- PubMed Search strategy and keywords**

| Total search strategy | #1 AND #2 AND #3 AND #4 |
| --- | --- |
| #1 | hospital*[MeSH Terms] |
| #2 | (((((((patient readmission[MeSH Terms]) ) OR (Recurrent admission*[Title/Abstract])) OR (readmission*[Title/Abstract])) OR (repeat admission*[Title/Abstract])) OR (frequent admission*[Title/Abstract])) OR (Second admission[Title/Abstract])) OR (return to hospital[Title/Abstract] OR (rehospitalisation*[Title/Abstract])) |
| #3 | ((((((((((((((((((((((((((((((((((telemedicine[MeSH Terms]) OR (tele-medicine[Title/Abstract])) OR (tele-nursing[Title/Abstract])) OR (tele-health[Title/Abstract])) OR (m-health[Title/Abstract])) OR (e-health[Title/Abstract])) OR (remote consultation[MeSH Terms])) OR (tele-consultation [Title/Abstract])) OR (digital intervention*[Title/Abstract])) OR (remote intervention*[Title/Abstract])) OR (telecare[Title/Abstract])) OR (tele-care[Title/Abstract])) (tele-monitoring[Title/Abstract])) OR (telemonitoring[Title/Abstract])) OR (teletherapy[Title/Abstract])) OR (tele-therapy[Title/Abstract])) OR (telepharmacy[Title/Abstract])) OR (tele-pharmacy[Title/Abstract])) OR (electronic prescription*[Title/Abstract])) OR (electronic prescribing[Title/Abstract])) OR (electronic pharmacy[Title/Abstract])) OR (remote pharmacy[Title/Abstract])) OR (digital pharmacy[Title/Abstract])) OR (digital health[Title/Abstract])) OR (digital medicine[Title/Abstract])) OR (digital nursing[Title/Abstract])) OR (emedicine[Title/Abstract])) OR (e-medicine[Title/Abstract])) OR (virtual nursing[Title/Abstract])) OR (remote nursing[Title/Abstract])) OR (virtual healthcare[Title/Abstract])) OR (remote healthcare[Title/Abstract])) OR (telemedicine robot[Title/Abstract])) OR (mobile health application[Title/Abstract])) OR (rural health nursing[Title/Abstract]) |
| #4 | (((((((((((drug therapy[MeSH Terms]) OR (hospital pharmacy service[MeSH Terms])) OR (drug information service[MeSH Terms])) OR (medication therapy management[MeSH Terms])) OR (medication system[MeSH Terms])) OR (drug utilization review[MeSH Terms])) OR (medication*[Title/Abstract])) OR (medication therapy[Title/Abstract])) OR (clinical pharmacology[Title/Abstract])) OR (clinical pharmacy[Title/Abstract])) OR (drug utilisation review[Title/Abstract])) OR (medication management[Title/Abstract]) |

**Table D-** **Scopus search strategy and keywords**

| Total search strategy | #1 AND #2 AND #3 AND #4 |
| --- | --- |
| #1 | TITLE-ABS-KEY (hospital*) |
| #2 | ( ( TITLE-ABS-KEY ( "recurrent admission*" ) OR TITLE-ABS-KEY ( readmission* ) OR TITLE-ABS-KEY ( "return to hospital" ) OR TITLE-ABS-KEY ( "repeat admission*" ) OR TITLE-ABS-KEY ( "frequent admission*" ) OR TITLE-ABS-KEY ( "second admission*" ) OR TITLE-ABS-KEY ( "patient readmission*" ) OR TITLE-ABS-KEY ( "rehospitalisation*" ) OR TITLE-ABS-KEY ( "rehospitalization*" ) OR TITLE-ABS-KEY ( "unplanned hospital readmission*" ) OR TITLE-ABS-KEY ( "hospital readmission*" ) ) ) |
| #3 | ( TITLE-ABS-KEY ( "digital intervention*" ) OR TITLE-ABS-KEY ( "remote intervention*" ) OR TITLE-ABS-KEY ( telecare ) OR TITLE-ABS-KEY ( tele-care ) OR TITLE-ABS-KEY ( telemonitoring ) OR TITLE-ABS-KEY ( tele-monitoring ) OR TITLE-ABS-KEY ( teletherapy ) OR TITLE-ABS-KEY ( tele-therapy ) OR TITLE-ABS-KEY ( telepharmacy ) OR TITLE-ABS-KEY ( tele-pharmacy ) OR TITLE-ABS-KEY ( telemedicine ) OR TITLE-ABS-KEY ( tele-medicine ) OR TITLE-ABS-KEY ( telenursing ) OR TITLE-ABS-KEY ( tele-nursing ) OR TITLE-ABS-KEY ( "virtual medicine" ) OR TITLE-ABS-KEY ( "mobile health" ) OR TITLE-ABS-KEY ( mhealth ) OR TITLE-ABS-KEY ( m-health ) OR TITLE-ABS-KEY ( telehealth ) OR TITLE-ABS-KEY ( tele-health ) OR TITLE-ABS-KEY ( ehealth ) OR TITLE-ABS-KEY ( e-health ) OR TITLE-ABS-KEY ( "remote consultation" ) OR TITLE-ABS-KEY ( "distance counselling" ) OR TITLE-ABS-KEY ( teleconsultation ) OR TITLE-ABS-KEY ( tele-consultation ) OR TITLE-ABS-KEY ( "electronic prescription*" ) OR TITLE-ABS-KEY ( "electronic prescribing" ) OR TITLE-ABS-KEY ( "electronic pharmacy" ) OR TITLE-ABS-KEY ( "remote pharmacy" ) OR TITLE-ABS-KEY ( "digital pharmacy" ) OR TITLE-ABS-KEY ( "digital health" ) OR TITLE-ABS-KEY ( "digital medicine" ) OR TITLE-ABS-KEY ( "digital nursing" ) OR TITLE-ABS-KEY ( emedicine ) OR TITLE-ABS-KEY ( e-medicine ) OR TITLE-ABS-KEY ( "virtual nursing" ) OR TITLE-ABS-KEY ( "remote nursing" ) OR TITLE-ABS-KEY ( "virtual healthcare" ) OR TITLE-ABS-KEY ( "remote healthcare" ) OR TITLE-ABS-KEY ( "telemedicine robot" ) OR TITLE-ABS-KEY ( "mobile health application" ) OR TITLE-ABS-KEY ( "rural health nursing" ) ) |
| #4 | ( TITLE-ABS-KEY ( medication* ) OR TITLE-ABS-KEY ( "medication management" ) OR TITLE-ABS-KEY ( "medication therapy management" ) OR TITLE-ABS-KEY ( "medication Therapy" ) OR TITLE-ABS-KEY ( "drug therapy" ) OR TITLE-ABS-KEY ( "medication reconciliation" ) OR TITLE-ABS-KEY ( "medication review" ) OR TITLE-ABS-KEY ( "hospital pharmacy service*" ) OR TITLE-ABS-KEY ( "clinical pharmacology" ) OR TITLE-ABS-KEY ( "clinical pharmacy" ) OR TITLE-ABS-KEY ( "drug information service*" ) OR TITLE-ABS-KEY ( "drug utilization review" ) OR TITLE-ABS-KEY ( "drug utilisation review" ) OR TITLE-ABS-KEY ( "medication system*" ) ) |

**Table E- Web of Science search strategy and keywords**

| Total search strategy | #1 AND #2 AND #3 AND #4 |
| --- | --- |
| #1 | TS=(hospital*) |
| #2 | ((((((((((( TS=( "recurrent admission*" )) OR TS=( readmission* )) OR TS=( "return to hospital" )) OR TS=( "repeat admission*" )) OR TS=( "frequent admission*" )) OR TS=( "second admission*" )) OR TS=( "patient readmission*" )) OR TS=( "rehospitalisation*" )) OR TS=( "rehospitalization*" )) OR TS=( "unplanned hospital readmission*" )) OR TS=( "hospital readmission*" )) |
| #3 | ((((((((((((((((((((((((((((((((((((((((((( TS=( "digital intervention*" )) OR TS=( "remote intervention*" )) OR TS=( telecare )) OR TS=( tele-care )) OR TS=( telemonitoring )) OR TS=( tele-monitoring )) OR TS=( teletherapy )) OR TS=( tele-therapy )) OR TS=( telepharmacy )) OR TS=( tele-pharmacy )) OR TS=( telemedicine )) OR TS=( tele-medicine )) OR TS=( telenursing )) OR TS=( tele-nursing )) OR TS=( "virtual medicine" )) OR TS=( "mobile health" )) OR TS=( mhealth )) OR TS=( m-health )) OR TS=( telehealth )) OR TS=( tele-health )) OR TS=( ehealth )) OR TS=( e-health )) OR TS=( "remote consultation" )) OR TS=( "distance counselling" )) OR TS=( teleconsultation )) OR TS=( tele-consultation )) OR TS=( "electronic prescription*" )) OR TS=( "electronic prescribing" )) OR TS=( "electronic pharmacy" )) OR TS=( "remote pharmacy" )) OR TS=( "digital pharmacy" )) OR TS=( "digital health" )) OR TS=( "digital medicine" )) OR TS=( "digital nursing" )) OR TS=( emedicine )) OR TS=( e-medicine )) OR TS=( "virtual nursing" )) OR TS=( "remote nursing" )) OR TS=( "virtual healthcare" )) OR TS=( "remote healthcare" )) OR TS=( "telemedicine robot" )) OR TS=( "mobile health application" )) OR TS=( "rural health nursing" )) |
| #4 | (((((((((((((( TS=( medication* )) OR TS=( "medication management" )) OR TS=( "medication therapy management" )) OR TS=( "medication Therapy" )) OR TS=( "drug therapy" )) OR TS=( "medication reconciliation" )) OR TS=( "medication review" )) OR TS=( "hospital pharmacy service*" )) OR TS=( "clinical pharmacology" )) OR TS=( "clinical pharmacy" )) OR TS=( "drug information service*" )) OR TS=( "drug utilization review" )) OR TS=( "drug utilisation review" )) OR TS=( "medication system*" )) |

**Table F- Proquest search strategy and keywords**

| Total search strategy | #1 AND #2 AND #3 AND #4 |
| --- | --- |
| #1 | Noft(hospital*) |
| #2 | NOFT( "recurrent admission*" ) OR NOFT( readmission* ) OR NOFT( "return to hospital" ) OR NOFT( "repeat admission*" ) OR NOFT( "frequent admission*" ) OR NOFT( "second admission*" ) OR NOFT( "patient readmission*" ) OR NOFT( "rehospitalisation*" ) OR NOFT( "rehospitalization*" ) OR NOFT( "unplanned hospital readmission*" ) OR NOFT( "hospital readmission*" ) |
| #3 | NOFT( "digital intervention*" ) OR NOFT( "remote intervention*" ) OR NOFT( telecare ) OR NOFT( tele-care ) OR NOFT( telemonitoring ) OR NOFT( tele-monitoring ) OR NOFT( teletherapy ) OR NOFT( tele-therapy ) OR NOFT( telepharmacy ) OR NOFT( tele-pharmacy ) OR NOFT( telemedicine ) OR NOFT( tele-medicine ) OR NOFT( telenursing ) OR NOFT( tele-nursing ) OR NOFT( "virtual medicine" ) OR NOFT( "mobile health" ) OR NOFT( mhealth ) OR NOFT( m-health ) OR NOFT( telehealth ) OR NOFT( tele-health ) OR NOFT( ehealth ) OR NOFT( e-health ) OR NOFT( "remote consultation" ) OR NOFT( "distance counselling" ) OR NOFT( teleconsultation ) OR NOFT( tele-consultation ) OR NOFT( "electronic prescription*" ) OR NOFT( "electronic prescribing" ) OR NOFT( "electronic pharmacy" ) OR NOFT( "remote pharmacy" ) OR NOFT( "digital pharmacy" ) OR NOFT( "digital health" ) OR NOFT( "digital medicine" ) OR NOFT( "digital nursing" ) OR NOFT( emedicine ) OR NOFT( e-medicine ) OR NOFT( "virtual nursing" ) OR NOFT( "remote nursing" ) OR NOFT( "virtual healthcare" ) OR NOFT( "remote healthcare" ) OR NOFT( "telemedicine robot" ) OR NOFT( "mobile health application" ) OR NOFT( "rural health nursing" ) |
| #4 | NOFT( "medication therapy management" ) OR NOFT( "medication Therapy" ) OR NOFT( "drug therapy" ) OR NOFT( "medication reconciliation" ) OR NOFT( "medication review" ) OR NOFT( "hospital pharmacy service*" ) OR NOFT( "clinical pharmacology" ) OR NOFT( "clinical pharmacy" ) OR NOFT( "drug information service*" ) OR NOFT( "drug utilization review" ) OR NOFT( "drug utilisation review" ) OR NOFT( "medication system*" ) |
