# Supplementary material for: Medication-focused telehealth interventions to reduce the hospital readmission rate: a systematic review
Source: J Pharm Policy Pract. 2025 Feb 5;18(1):2457411. doi: 10.1080/20523211.2025.2457411 (PMC11800339; doi:10.1080/20523211.2025.2457411)
Supplement: Supplemental Material S2 [file JPPP_A_2457411_SM0363.docx]

**Table G- Quality assessment summary of the included RCTs using ROB2 tool**

| Study authors | Antonicelli 2010 | |
| --- | --- | --- |
| Bias | **Judgement** | **Support for judgement** |
| **Randomisation process** | Some concern | The study was randomised while the randomisation method was not specified. No significant baseline difference between groups. |
| **Deviation from intended interventions** | Some concern | No information provided on the concealment of the group assignment |
| **Missing outcome data** | Low risk | No missing outcome data reported |
| **Measurement of the outcome** | Some concern | The method used for collecting hospital readmission rate data was self-report via telephone call. |
| **Selection of the reported result** | Some concern | Not described |
| **Overall quality assessment** | Some concern |  |
| Study authors | **Biese 2014** | |
| Bias | **Judgement** | **Support for judgement** |
| **Randomisation process** | Low risk | Computer-generated randomisation concealed from participants. No significant baseline difference between groups. |
| **Deviation from intended interventions** | High risk | The patients were blinded to group assignment, while healthcare providers and research assistants were not. No information was provided regarding the concealment of assessors. |
| **Missing outcome data** | High risk | Among 178 participants after the randomisation, 58 of them left the study due to multiple reasons at different stages of the study and just 120 participants included in the final analaysis. Reason for no folLow risk up is was unknown |
| **Measurement of the outcome** | Some concern | The method used for collecting hospital readmissionrate data was self-report via telephone call. |
| **Selection of the reported result** | Low risk | Results all reported according to data analysis plan and outcomes |
| **Overall quality assessment** | High risk |  |
| Study authors | **Biese 2018** | |
| Bias | **Judgement** | **Support for judgement** |
| **Randomisation process** | Low risk | Block randomisation was implemented and all investigators except for the statistician and nursed were blinded. Also based on the participant demograpic characteristics of each group, there were most likely no significant baseline differences either. |
| **Deviation from intended interventions** | Low risk | Participants and investigators were blind to study except the statisticians and calling nurses. |
| **Missing outcome data** | Low risk | Nearly 98 of the randomized participants stayed in the study.  3.1 continuous outcomes, availability of data from 95% of the participants will often be sufficient. |
| **Measurement of the outcome** | Some concern | The method used for collecting hospital readmissionrate data was self-report via telephone call. |
| **Selection of the reported result** | Low risk | Results all reported according to data analysis plan and outcomes |
| **Overall quality assessment** | Some concern |  |
| Study authors | **Boockvar 2022** | |
| Bias | **Judgement** | **Support for judgement** |
| **Randomisation process** | Low risk | Computer-generated randomisation was stated. Baseline characteristics were also balanced among the study groups. |
| **Deviation from intended interventions** | High risk | Care team assigned the participants to control and intervention group |
| **Missing outcome data** | High risk | Almost 57% of randomised participants excluded from the study ant no clear reason was explained. |
| **Measurement of the outcome** | Low risk | Outcome measures were collected by trained research assistants from electronic health records. |
| **Selection of the reported result** | Low risk | Results all reported according to data analysis plan and outcomes |
| **Overall quality assessment** | High risk |  |
| Study authors | **Broadbent 2018** | |
| Bias | **Judgement** | **Support for judgement** |
| **Randomisation process** | Some concern | Randomisation via a randomisation program using number sequence was implemented by a statistician in a separate location. Intervention group was indicated to have more hospitalisations in the past and comorbidities which had the potential in affecting the study results. |
| **Deviation from intended interventions** | Low risk | Both participants and inventors were blind to the th group assignment. |
| **Missing outcome data** | Some concern | Nearly 85% of the randomized participants included in final analysis The study was small scale and is a pilot. |
| **Measurement of the outcome** | Low risk | The rate of hospital readmission was reported by robot. |
| **Selection of the reported result** | Low risk | Results all reported according to data analysis plan and outcomes |
| **Overall quality assessment** | Some concern |  |
| Study authors | **Casida 2022** | |
| Bias | **Judgement** | **Support for judgement** |
| **Randomisation process** | Low risk | Computer-generated randomisation concealed from statistician. No significant baseline difference between groups. |
| **Deviation from intended interventions** | Some concern | No information on the concealment of group assignment to participants and healthcare providers. |
| **Missing outcome data** | High risk | Only 80% of the randomized participants included in final analysis |
| **Measurement of the outcome** | Low risk | Data on hospital readmission rate was collected through interview with both patients and healthcare providers. |
| **Selection of the reported result** | Some concern | Not described |
| **Overall quality assessment** | High risk |  |
| Study authors | **Chen 2018** | |
| Bias | **Judgement** | **Support for judgement** |
| **Randomisation process** | Some concern | The study was randomised while the randomisation method was not specified. No significant baseline difference between groups. |
| **Deviation from intended interventions** | Low risk | Both participants and inventors were blind to the th group assignment. |
| **Missing outcome data** | Low risk | More than 95% of the randomized participants included in final analysis |
| **Measurement of the outcome** | Some concern | The method used for collecting hospital readmission rate data was self-report via telephone call. |
| **Selection of the reported result** | Low risk | Results all reported according to data analysis plan and outcomes |
| **Overall quality assessment** | Some concern |  |
| Study authors | **Dar 2009** | |
| Bias | **Judgement** | **Support for judgement** |
| **Randomisation process** | Low risk | Analysis performed by blinded researchers. Actual randomisation was down through computerisation. Baseline characteristics were also stated to be similar. |
| **Deviation from intended interventions** | Low risk | Both participants and inventors were blind to the th group assignment. |
| **Missing outcome data** | Low risk | No missing data |
| **Measurement of the outcome** | Some concern | The method used for collecting hospital readmission rate data was self-report |
| **Selection of the reported result** | Low risk | Results all reported according to data analysis plan and outcomes |
| **Overall quality assessment** | Some concern |  |
| Study authors | **Devito 2016** | |
| Bias | **Judgement** | **Support for judgement** |
| **Randomisation process** | Some concern | Computer generated randomisation The baseline information of the control and intervention group was not consistent. |
| **Deviation from intended interventions** | Low risk | Both participants and inventors were blind to the th group assignment. |
| **Missing outcome data** | Low risk | No missing data |
| **Measurement of the outcome** | Low risk | hospital readmission outcomes were abstracted from the medical records. |
| **Selection of the reported result** | Low risk | Results all reported according to data analysis plan and outcomes |
| **Overall quality assessment** | Some concern |  |
| Study authors | **Dhalla 2014** | |
| Bias | **Judgement** | **Support for judgement** |
| **Randomisation process** | Low risk | Computer-generated randomisation was concealed from participants. No important between-group differences either. |
| **Deviation from intended interventions** | Some concern | Both participants and inventors were aware of the th group assignment. Due to the nature of study was impossible to conceal the assignment |
| **Missing outcome data** | Low risk | Only less than 1% of randomized participants exluded from the study |
| **Measurement of the outcome** | Low risk | Hospital readmission outcomes were abstracted from the medical records. |
| **Selection of the reported result** | Low risk | Results all reported according to data analysis plan and outcomes |
| **Overall quality assessment** | Some concern |  |
| Study authors | **Gallagher2016** | |
| Bias | **Judgement** | **Support for judgement** |
| **Randomisation process** | Low risk | Computer-generated randomisation was concealed from participants. No significant differences found between the different groups at baseline. |
| **Deviation from intended interventions** | Some concern | No information on the blindess of the trial. |
| **Missing outcome data** | High risk | 10% missing, |
| **Measurement of the outcome** | Low risk | Hospital readmission outcomes were abstracted from the medical records. |
| **Selection of the reported result** | Low risk | Results all reported according to data analysis plan and outcomes |
| **Overall quality assessment** | High risk |  |
| Study authors | **Goldman 2014** | |
| Bias | **Judgement** | **Support for judgement** |
| **Randomisation process** | Low risk | Computer-generated randomisation No significant differences found between the different groups at baseline. |
| **Deviation from intended interventions** | Some concern | Only healthcare providers were not blind to group assignment. |
| **Missing outcome data** | Low risk | Almost 90% of randomised participants were included in the analysis. Missing data was handled through sensitivity analyses |
| **Measurement of the outcome** | Low risk | Hospital readmission outcomes were abstracted from the medical records. |
| **Selection of the reported result** | Low risk | Results all reported according to data analysis plan and outcomes |
| **Overall quality assessment** | Some concern |  |
| Study authors | **Habib 2021** | |
| Bias | **Judgement** | **Support for judgement** |
| **Randomisation process** | Low risk | Computer-generated randomisation was concealed from participants. No significant baseline difference between groups. |
| **Deviation from intended interventions** | Low risk | Both participants and inventors were blind to the th group assignment. |
| **Missing outcome data** | Low risk | Almost 75% of randomised participants were included in the analysis. Missing data was justified |
| **Measurement of the outcome** | Low risk | Hospital readmission outcomes were abstracted from the medical records. |
| **Selection of the reported result** | Some concern | No data analysis plan to determine whether any analyses have been omitted in the results |
| **Overall quality assessment** | Some concern |  |
| Study authors | **Hale 2016** | |
| Bias | **Judgement** | **Support for judgement** |
| **Randomisation process** | Some concern | The study was randomised while the randomisation method was not specified. No significant baseline difference between groups. |
| **Deviation from intended interventions** | Some concern | No information on the concealment of the group assignment from participants and investigators. |
| **Missing outcome data** | Some concern | Almost 87% of randomised participants were included in the analysis. Missing data was justified |
| **Measurement of the outcome** | Low risk | Hospital readmission outcomes were abstracted from the medical records. |
| **Selection of the reported result** | Low risk | Results all reported according to data analysis plan and outcomes |
| **Overall quality assessment** | Some concern |  |
| Study authors | **Jerant 2003** | |
| Bias | **Judgement** | **Support for judgement** |
| **Randomisation process** | Some concern | The baseline information of the control and intervention group was not consistent. There was a trend towards significance (p = 0.0666) for the difference between groups in mean duration of CHF at the time of study. |
| **Deviation from intended interventions** | Some concern | No information on the concealment of the group assignment from participants and investigators |
| **Missing outcome data** | Low risk | No missing data |
| **Measurement of the outcome** | Low risk | Outcomes were abstracted from the medical records. |
| **Selection of the reported result** | Some concern | No data analysis plan to determine whether any analyses have been omitted in the results |
| **Overall quality assessment** | Some concern |  |
| Study authors | **Liang 2021** | |
| Bias | **Judgement** | **Support for judgement** |
| **Randomisation process** | Low risk | Computer-generated randomisation. No significant between-group differencesin baseline characteristics. |
| **Deviation from intended interventions** | Some concern | No information on the concealment of the group assignment from participants and investigators. |
| **Missing outcome data** | Low risk | No missing data |
| **Measurement of the outcome** | Low risk | Outcomes were abstracted from the medical records. |
| **Selection of the reported result** | Low risk | Results all reported according to data analysis plan and outcomes |
| **Overall quality assessment** | Some concern |  |
| Study authors | **Noel 2020** | |
| Bias | **Judgement** | **Support for judgement** |
| **Randomisation process** | Some concern | Computer-generated randomisation was not concealed from participants. No significant baseline difference between groups. |
| **Deviation from intended interventions** | Some concern | No information on the concealment of the group assignment from participants and investigators. |
| **Missing outcome data** | High risk | Almost 75% of randomised participants were included but the missing data was not justified |
| **Measurement of the outcome** | Low risk | Hospital readmission outcomes were abstracted from the medical records. |
| **Selection of the reported result** | Some concern | Not described |
| **Overall quality assessment** | High risk |  |
| Study authors | **Piette 2021** | |
| Bias | **Judgement** | **Support for judgement** |
| **Randomisation process** | Low risk | Computer-generated randomisation was concealed from participants. No baseline differences detected between groups. |
| **Deviation from intended interventions** | Some concern | No information on the concealment of the group assignment from participants and investigators. |
| **Missing outcome data** | Low risk | No missing data, check the consort diagram |
| **Measurement of the outcome** | Low risk | Outcomes were abstracted from the medical records. |
| **Selection of the reported result** | Low risk | Results all reported according to data analysis plan and outcomes |
| **Overall quality assessment** | Some concern |  |
| Study authors | **Schmaderer 2022** | |
| Bias | **Judgement** | **Support for judgement** |
| **Randomisation process** | Some concern | Computer-generated randomisation was not concealed from participants. No significant baseline difference between groups. |
| **Deviation from intended interventions** | Some concern | No information on the concealment of the group assignment from participants and investigators. |
| **Missing outcome data** | Low risk | Almost 94% of randomised participants were included in the analysis. |
| **Measurement of the outcome** | Some concern | The method used for collecting hospital readmission rate data was self-report via telephone call. |
| **Selection of the reported result** | Low risk | Results all reported according to data analysis plan and outcomes |
| **Overall quality assessment** | Some concern |  |
| Study authors | **TulepberGenov 2022** | |
| Bias | **Judgement** | **Support for judgement** |
| **Randomisation process** | Some concern | The study was randomised while the randomisation method was not specified. |
| **Deviation from intended interventions** | Some concern | No information on the concealment of the group assignment from participants and investigators. |
| **Missing outcome data** | High risk | Almost 46% of randomised participants were included in analysis but the missing data was not justified. |
| **Measurement of the outcome** | Low risk | A Cox proportional hazards model to account for the clustering effect, adjusted for baseline risk factors, was used to analyse readmission. |
| **Selection of the reported result** | Low risk | Results all reported according to data analysis plan and outcomes. |
| **Overall quality assessment** | High risk |  |
| Study authors | **Valimaki 2017** | |
| Bias | **Judgement** | **Support for judgement** |
| **Randomisation process** | Low risk | Computer-generated randomisation was concealed from participants. No significant baseline difference between groups. |
| **Deviation from intended interventions** | Low risk | Both participants and inventors were blind to the group assignment. |
| **Missing outcome data** | Some concern | Although the intention to treat analysis was conducted, data on about 40% of the participants was not available. |
| **Measurement of the outcome** | Low risk | HOSPITAL READMISSION outcomes were abstracted from the medical records. |
| **Selection of the reported result** | Some concern | Not described |
| **Overall quality assessment** | Some concern |  |
| Study authors | **Widmer 2017** | |
| Bias | **Judgement** | **Support for judgement** |
| **Randomisation process** | Low risk | Computer-generated randomisation was concealed from participants. No significant baseline difference between groups. |
| **Deviation from intended interventions** | Some concern | The patients were blinded to group assignment, while healthcare providers were not. |
| **Missing outcome data** | High risk | Almost 90% of randomised participants were included in the analysis with no justification. |
| **Measurement of the outcome** | Low risk | HOSPITAL READMISSION outcomes were abstracted from the medical records. |
| **Selection of the reported result** | Low risk | Results all reported according to data analysis plan and outcomes |
| **Overall quality assessment** | High risk |  |
| Study authors | **Zhang 2019** | |
| Bias | **Judgement** | **Support for judgement** |
| **Randomisation process** | Low risk | Randomisation number table method. No significant baseline difference between groups. |
| **Deviation from intended interventions** | Some concern | No information on the concealment of the group assignment from participants and investigators. |
| **Missing outcome data** | Low risk | No missing data |
| **Measurement of the outcome** | Low risk | HOSPITAL READMISSION outcomes were abstracted from the medical records. |
| **Selection of the reported result** | Low risk | Results all reported according to data analysis plan and outcomes |
| **Overall quality assessment** | Some concern |  |
