# Supplementary material for: Medication-focused telehealth interventions to reduce the hospital readmission rate: a systematic review
Source: J Pharm Policy Pract. 2025 Feb 5;18(1):2457411. doi: 10.1080/20523211.2025.2457411 (PMC11800339; doi:10.1080/20523211.2025.2457411)
Supplement: Supplemental Material S3 [file JPPP_A_2457411_SM0362.docx]

**Table S3. Intervention Complexity assessment summary of the included RCTs**

| **Study authors:** Antonicelli *et al.,* 2010, Italy | | | |  |  |  |
| --- | --- | --- | --- | --- | --- | --- |
| **Complexity** | **Score** | **Judgement** | **Support for judgement** |  |  |  |
| **1-Description of the Active components included in the intervention** | 3 | More than one Component as a bundle, | Vital sign monitoring, ECG monitoring, Symptoms monitoring, medication adherence support, medication managment |  |  |  |
| **2-Description of Behaviour or actions of intervention recipients or participants to which the intervention is directed** | 1 | Single target | Medication adherence |  |  |  |
| **3-Description of Organisational levels and categories targeted by the intervention** | 1 | Single Category | Patients only |  |  |  |
| **4-The degree of tailoring intended or flexibility permitted across sites or individuals in applying or implementing the intervention** | 2 | Moderately tailored/flexible, | Modification of therapeutic regimen and medication advice for each patient |  |  |  |
| **5-Description of The level of skill required by those delivering the intervention in order to meet the intervention’s objectives** | 3 | High level skills | CHF team trained for home telemonitoring |  |  |  |
| **6-Description of the The level of skill required for the targeted behaviour when entering the included studies by those receiving the intervention, in order to meet the intervention’s objectives** | 1 | Basic skills | No specific skill required |  |  |  |
| **Overall score** | **11** | **Medium complexity** |  |  |  |  |
| **Study authors:** Biese *et al*., 2014, USA | | | |  |  |  |
| **Complexity** | **Score** | **Judgement** | **Support for judgement** |  |  |  |
| **1-Description of the Active components included in the intervention** | 3 | More than one Component as a bundle | Post-discharge review, Follow-up reinforcement, Health status advice, medication adherence support, medication managment |  |  |  |
| **2-Description of Behaviour or actions of intervention recipients or participants to which the intervention is directed** | 2 | Dual-target | Medication adherence and follow up appointmnets with doctors |  |  |  |
| **3-Description of Organisational levels and categories targeted by the intervention** | 1 | Single category | Patients |  |  |  |
| **4-The degree of tailoring intended or flexibility permitted across sites or individuals in applying or implementing the intervention** | 2 | Moderately tailored/flexible | Patient-specific instructions implemented in calls |  |  |  |
| **5-Description of The level of skill required by those delivering the intervention in order to meet the intervention’s objectives** | 2 | Intermediate skills | Study nurse trained for conducting follow up interviews |  |  |  |
| **6-Description of the The level of skill required for the targeted behaviour when entering the included studies by those receiving the intervention, in order to meet the intervention’s objectives** | 1 | Basic skills | No specified skills require by participants |  |  |  |
| **Overall score** | **11** | **Medium complexity** |  |  |  |  |
| **Study authors:** Biese *et al.,* 2018, USA | | | |  |  |  |
| **Complexity** | **Score** | **Judgement** | **Support for judgement** |  |  |  |
| **1-Description of the Active components included in the intervention** | 3 | More than one Component as a bundle | Post-discharge review, Follow-up reinforcement, Health status advice, medication adherence support, medication managment |  |  |  |
| **2-Description of Behaviour or actions of intervention recipients or participants to which the intervention is directed** | 2 | Dual-target | Medication adherence and follow up appointmnets with doctors |  |  |  |
| **3-Description of Organisational levels and categories targeted by the intervention** | 1 | Single category | Patients only |  |  |  |
| **4-The degree of tailoring intended or flexibility permitted across sites or individuals in applying or implementing the intervention** | 2 | Moderately tailored/flexible | Advice offering depending on patient circumstances |  |  |  |
| **5-Description of The level of skill required by those delivering the intervention in order to meet the intervention’s objectives** | 2 | Intermediate skills | Nurses trained in telephonic medicine and triage and received additional training for this study |  |  |  |
| **6-Description of the The level of skill required for the targeted behaviour when entering the included studies by those receiving the intervention, in order to meet the intervention’s objectives** | 1 | Basic skills | No specified skills require by participants |  |  |  |
| **Overall score** | **12** | **High complexity** |  |  |  |  |
| **Study authors:** Boockvar *et al*., 2022, USA | | | |  |  |  |
| **Complexity** | **Score** | **Judgement** | **Support for judgement** |  |  |  |
| **1-Description of the Active components included in the intervention** | 3 | More than one Component as a bundle | Education on condition, Symptoms monitoring, medication adherence |  |  |  |
| **2-Description of Behaviour or actions of intervention recipients or participants to which the intervention is directed** | 3 | Multi-target | Self-management of taking medication, symptoms checking and booking the follow up appontments with doctor |  |  |  |
| **3-Description of Organisational levels and categories targeted by the intervention** | 1 | Single category | Patients only |  |  |  |
| **4-The degree of tailoring intended or flexibility permitted across sites or individuals in applying or implementing the intervention** | 2 | Moderately tailored/flexible | Patient-specific education |  |  |  |
| **5-Description of The level of skill required by those delivering the intervention in order to meet the intervention’s objectives** | 2 | Intermediate skills | Social workers trained to conduct the interventions. |  |  |  |
| **6-Description of the The level of skill required for the targeted behaviour when entering the included studies by those receiving the intervention, in order to meet the intervention’s objectives** | 1 | Basic skills | No specified skills require by participants |  |  |  |
| **Overall score** | **12** | **High complexity** |  |  |  |  |
| **Study authors:** Broadbent *et al.,* 2018, NZ | | | |  |  |  |
| **Complexity** | **Score** | **Judgement** | **Support for judgement** |  |  |  |
| **1-Description of the Active components included in the intervention** | 3 | More than one Component as a bundle | Vital sign monitoring, Symptoms monitoring, Exercise reminder, Education on condition, medication adherence support |  |  |  |
| **2-Description of Behaviour or actions of intervention recipients or participants to which the intervention is directed** | 3 | Multi-target | Exercise , Medication adherence, knowledge on symptoms for self-care |  |  |  |
| **3-Description of Organisational levels and categories targeted by the intervention** | 1 | Single category | Patients only |  |  |  |
| **4-The degree of tailoring intended or flexibility permitted across sites or individuals in applying or implementing the intervention** | 3 | Highly tailored/flexible | Patient-specific reponses |  |  |  |
| **5-Description of the level of skill required by those delivering the intervention in order to meet the intervention’s objectives** | 2 | Intermediate skills | Healthcare team trained for using robot |  |  |  |
| **6-Description of the The level of skill required for the targeted behaviour when entering the included studies by those receiving the intervention, in order to meet the intervention’s objectives** | 2 | Intermediate skills | Particpants trained for using robot |  |  |  |
| **Overall score** | **13** | **High complexity** |  |  |  |  |
| **Study authors:** Casida *et al*., 2022, USA | | | |  |  |  |
| **Complexity** | **Score** | **Judgement** | **Support for judgement** |  |  |  |
| **1-Description of the Active components included in the intervention** | 3 | More than one Component as a bundle | Education on condition, Vital sign monitoring, Symptoms monitoring, Dietary and fluid intake monitoring, Blood test monitoring, medication adherence and medication managment |  |  |  |
| **2-Description of Behaviour or actions of intervention recipients or participants to which the intervention is directed** | 3 | Multi-target | Diet and fluid intak, Madication adherence, knowledge on sysmptoms, record-keeping |  |  |  |
| **3-Description of Organisational levels and categories targeted by the intervention** | 1 | Single category | Patients only |  |  |  |
| **4-The degree of tailoring intended or flexibility permitted across sites or individuals in applying or implementing the intervention** | 3 | Highly tailored/flexible | Assessments done based on patient-specific abnormalities |  |  |  |
| **5-Description of The level of skill required by those delivering the intervention in order to meet the intervention’s objectives** | 2 | Intermediate skills | Nurses trained on using VAD Care App |  |  |  |
| **6-Description of the The level of skill required for the targeted behaviour when entering the included studies by those receiving the intervention, in order to meet the intervention’s objectives** | 1 | Basic skills | No specified skills require by participants |  |  |  |
| **Overall score** | **13** | **High complexity** |  |  |  |  |
| **Study authors:** Chen *et al.,* 2019, China | | | |  |  |  |
| **Complexity** | **Score** | **Judgement** | **Support for judgement** |  |  |  |
| **1-Description of the Active components included in the intervention** | 3 | More than one Component as a bundle | Education on condition, Lifestyle education, Follow-up reinforcement, medication adherence support |  |  |  |
| **2-Description of Behaviour or actions of intervention recipients or participants to which the intervention is directed** | 3 | Multi-target | Lifestyle, medication adherence, knowledge on symptoms for self-care |  |  |  |
| **3-Description of Organisational levels and categories targeted by the intervention** | 1 | Single category | Patients only |  |  |  |
| **4-The degree of tailoring intended or flexibility permitted across sites or individuals in applying or implementing the intervention** | 1 | Inflexible | Messages were all standardised and scheduled |  |  |  |
| **5-Description of The level of skill required by those delivering the intervention in order to meet the intervention’s objectives** | 1 | Basic skills | Messages were sent, no specialised training needed |  |  |  |
| **6-Description of the The level of skill required for the targeted behaviour when entering the included studies by those receiving the intervention, in order to meet the intervention’s objectives** | 1 | Basic skills | No specified skills require by consumer |  |  |  |
| **Overall score** | **10** | **Medium complexity** |  |  |  |  |
| **Study authors:** Dar *et al*., 2009, UK | | | |  |  |  |
| **Complexity** | **Score** | **Judgement** | **Support for judgement** |  |  |  |
| **1-Description of the Active components included in the intervention** | 3 | More than one Component as a bundle | Vital sign monitoring, Lifestyle education, medication adherence support, medication management |  |  |  |
| **2-Description of Behaviour or actions of intervention recipients or participants to which the intervention is directed** | 3 | Multi-target | Lifestyle, medication adherence knowledge on symptoms  Daily check of weight and blood pressure, |  |  |  |
| **3-Description of Organisational levels and categories targeted by the intervention** | 1 | Single category | Patients only |  |  |  |
| **4-The degree of tailoring intended or flexibility permitted across sites or individuals in applying or implementing the intervention** | 2 | Moderately tailored/flexible | Telephone calls prompted upon patient-specific abnormalities |  |  |  |
| **5-Description of The level of skill required by those delivering the intervention in order to meet the intervention’s objectives** | 2 | Intermediate skills | Team Trained on using Honeywell HomMed |  |  |  |
| **6-Description of the The level of skill required for the targeted behaviour when entering the included studies by those receiving the intervention, in order to meet the intervention’s objectives** | 1 | Basic skills | No specified skills require by consumer |  |  |  |
| **Overall score** | 12 | **High complexity** |  |  |  |  |
| **Study authors:** DeVito Dabbs *et al.,* 2016, USA | | | |  |  |  |
| **Complexity** | **Score** | **Judgement** | **Support for judgement** |  |  |  |
| **1-Description of the Active components included in the intervention** | 3 | More than one Component as a bundle | Multiple components (recording daily health indicators, receive feedback messages,medication adherence support etc.) implemented as a bundle via Pocket PATH |  |  |  |
| **2-Description of Behaviour or actions of intervention recipients or participants to which the intervention is directed** | 1 | Single target | Medication adherence |  |  |  |
| **3-Description of Organisational levels and categories targeted by the intervention** | 1 | Single category | Patients |  |  |  |
| **4-The degree of tailoring intended or flexibility permitted across sites or individuals in applying or implementing the intervention** | 2 | Moderately tailored/flexible | Feedback messages sent based on patient-specific abnormalities |  |  |  |
| **5-Description of The level of skill required by those delivering the intervention in order to meet the intervention’s objectives** | 2 | Intermediate skills | Team trained on using Pocket PATH |  |  |  |
| **6-Description of the The level of skill required for the targeted behaviour when entering the included studies by those receiving the intervention, in order to meet the intervention’s objectives** | 1 | Basic skills | No specified skills require by participant |  |  |  |
| **Overall score** | 10 | **Medium complexity** |  |  |  |  |
| **Study authors:** Dhalla *et al.,* 2014, Canada | | | |  |  |  |
| **Complexity** | **Score** | **Judgement** | **Support for judgement** |  |  |  |
| **1-Description of the Active components included in the intervention** | 3 | More than one Component as a bundle | Post-discharge interventions delivered as virtual ward |  |  |  |
| **2-Description of Behaviour or actions of intervention recipients or participants to which the intervention is directed** | NA |  |  |  |  |  |
| **3-Description of Organisational levels and categories targeted by the intervention** | 1 | Single category | Patients only |  |  |  |
| **4-The degree of tailoring intended or flexibility permitted across sites or individuals in applying or implementing the intervention** | 3 | Highy tailored\flexible | Individualised management plans |  |  |  |
| **5-Description of The level of skill required by those delivering the intervention in order to meet the intervention’s objectives** | 1 | Basic skills | Interprofessional team with no extra training |  |  |  |
| **6-Description of the The level of skill required for the targeted behaviour when entering the included studies by those receiving the intervention, in order to meet the intervention’s objectives** | 1 | Basic skills | No specified skills require by participant |  |  |  |
| **Overall score** | 9 | **Medium complexity** |  |  |  |  |
| **Study authors:** Gallagher *et al.,* 2017, USA | | | |  |  |  |
| **Complexity** | **Score** | **Judgement** | **Support for judgement** |  |  |  |
| **1-Description of the Active components included in the intervention** | 1 | One component | Medication adherence support |  |  |  |
| **2-Description of Behaviour or actions of intervention recipients or participants to which the intervention is directFed** | 1 | Single target | Medication adherence |  |  |  |
| **3-Description of Organisational levels and categories targeted by the intervention** | 1 | Single category | Patients |  |  |  |
| **4-The degree of tailoring intended or flexibility permitted across sites or individuals in applying or implementing the intervention** | 2 | Moderately tailored/flexible | Actual implementation depended on patient adherence |  |  |  |
| **5-Description of The level of skill required by those delivering the intervention in order to meet the intervention’s objectives** | 1 | Basic skills | Social worker with no extra training |  |  |  |
| **6-Description of the The level of skill required for the targeted behaviour when entering the included studies by those receiving the intervention, in order to meet the intervention’s objectives** | 1 | Basic skills | No specified skills require by participant |  |  |  |
| **Overall score** | 7 | **Medium complexity** |  |  |  |  |
| **Study authors:** Goldman *et al.,* 2014, USA | | | |  |  |  |
| **Complexity** | **Score** | **Judgement** | **Support for judgement** |  |  |  |
| **1-Description of the Active components included in the intervention** | 3 | More than one Component as a bundle | Education on condition, Follow-up reinforcement, post-discharge review, medication adherence support, medication management |  |  |  |
| **2-Description of Behaviour or actions of intervention recipients or participants to which the intervention is directed** | 3 | Multi-target | Medication adherence, booking follow-up appointment with doctors, knowledge on condition for self-care |  |  |  |
| **3-Description of Organisational levels and categories targeted by the intervention** | 1 | Single category | Patients |  |  |  |
| **4-The degree of tailoring intended or flexibility permitted across sites or individuals in applying or implementing the intervention** | 2 | Moderately tailored/flexible | Patient-specific education |  |  |  |
| **5-Description of The level of skill required by those delivering the intervention in order to meet the intervention’s objectives** | 2 | Intermediate skills | Registered nurses and nurse practitioner received ongoing training |  |  |  |
| **6-Description of the The level of skill required for the targeted behaviour when entering the included studies by those receiving the intervention, in order to meet the intervention’s objectives** | 1 | Basic skills | No specified skills require by participant |  |  |  |
| **Overall score** | 12 | **High complexity** |  |  |  |  |
| **Study authors:** Habib *et al.,* 2021, Canada | | | |  |  |  |
| **Complexity** | **Score** | **Judgement** | **Support for judgement** |  |  |  |
| **1-Description of the Active components included in the intervention** | 3 | More than one Component as a bundle | Medication education, adherence alerts, interaction sheckers implemented as a bundle via SAM mobile app |  |  |  |
| **2-Description of Behaviour or actions of intervention recipients or participants to which the intervention is directed** | 1 | Single target | Medication adherence |  |  |  |
| **3-Description of Organisational levels and categories targeted by the intervention** | 1 | Single category | Patients |  |  |  |
| **4-The degree of tailoring intended or flexibility permitted across sites or individuals in applying or implementing the intervention** | 2 | Moderately tailored/flexible | Patient-specific information tailored on app |  |  |  |
| **5-Description of The level of skill required by those delivering the intervention in order to meet the intervention’s objectives** | 2 | Intermediate skills | Nurses received ongoing training |  |  |  |
| **6-Description of the The level of skill required for the targeted behaviour when entering the included studies by those receiving the intervention, in order to meet the intervention’s objectives** | 1 | Basic skills | No specified skills require by participant |  |  |  |
| **Overall score** | 12 | **High complexity** |  |  |  |  |
| **Study authors:** Hale *et al.,* 2016, USA | | | |  |  |  |
| **Complexity** | **Score** | **Judgement** | **Support for judgement** |  |  |  |
| **1-Description of the Active components included in the intervention** | 3 | More than one Component as a bundle | Medication monitoring device and phone call for medication reconciliation |  |  |  |
| **2-Description of Behaviour or actions of intervention recipients or participants to which the intervention is directed** | 1 | Single target | Medication adherence |  |  |  |
| **3-Description of Organisational levels and categories targeted by the intervention** | 1 | Single category | Patients |  |  |  |
| **4-The degree of tailoring intended or flexibility permitted across sites or individuals in applying or implementing the intervention** | 2 | Moderately tailored/flexible | Patient-specific information tailored on app |  |  |  |
| **5-Description of The level of skill required by those delivering the intervention in order to meet the intervention’s objectives** | 1 | Basic | No specified skills require by nurses |  |  |  |
| **6-Description of the The level of skill required for the targeted behaviour when entering the included studies by those receiving the intervention, in order to meet the intervention’s objectives** | 2 | Intermediate skills | Patients received training to use the device |  |  |  |
| **Overall Score** | 10 | **Medium complexity** |  |  |  |  |
|  |  |  |  |  |  |  |
| **Study authors:** Jerant *et al.,* 2003, | | | | **Score** | **Judgement** | **Support for judgement** |
| **Complexity** | **Score** | **Judgement** | **Support for judgement** |  |  |  |
| **1-Description of the Active components included in the intervention** | 3 | More than one Component as a bundle | Medication monitoring device and phone call for medication reconciliation |  |  |  |
| **2-Description of Behaviour or actions of intervention recipients or participants to which the intervention is directed** | 3 | Multi-target | Medication adherence, coping skills, lifestyle |  |  |  |
| **3-Description of Organisational levels and categories targeted by the intervention** | 1 | Single category | Patients |  |  |  |
| **4-The degree of tailoring intended or flexibility permitted across sites or individuals in applying or implementing the intervention** | 2 | Moderately tailored/flexible | Emergency calls can be made by patients |  |  |  |
| **5-Description of The level of skill required by those delivering the intervention in order to meet the intervention’s objectives** | 1 | Basic | No specified skills require by nurses |  |  |  |
| **6-Description of the The level of skill required for the targeted behaviour when entering the included studies by those receiving the intervention, in order to meet the intervention’s objectives** | 1 | Basic skills | No specified skills require by participants |  |  |  |
| **Overall score** | 11 | **Medium complexity** |  |  |  |  |
| **Study authors:** Noel *et al*. 2020, USA | | | |  |  |  |
| **Complexity** | **Score** | **Judgement** | **Support for judgement** |  |  |  |
| **1-Description of the Active components included in the intervention** | 3 | More than one Component as a bundle | Vital sign monitoring, medication adherence support and medication managment |  |  |  |
| **2-Description of Behaviour or actions of intervention recipients or participants to which the intervention is directed** | 2 | Dual target | Medication adherence, knowledge on condition and symsptoms for self-care |  |  |  |
| **3-Description of Organisational levels and categories targeted by the intervention** | 1 | Single category | Patients |  |  |  |
| **4-The degree of tailoring intended or flexibility permitted across sites or individuals in applying or implementing the intervention** | 2 | Moderately tailored/flexible |  |  |  |  |
| **5-Description of The level of skill required by those delivering the intervention in order to meet the intervention’s objectives** | 2 | Intermediate skills | Physicians were trained to ask open-ended questions to assess medications taken, as well as perform validation of patient histories using Electronic Medical Record (EMR) and pharmacy data. |  |  |  |
| **6-Description of the The level of skill required for the targeted behaviour when entering the included studies by those receiving the intervention, in order to meet the intervention’s objectives** | 1 | Basic skills | No specified skills require by participant |  |  |  |
| **Overall score** | 11 | **Medium complexity** |  |  |  |  |
| **Study authors:** Piette *et al*., 2021, USA | | | |  |  |  |
| **Complexity** | **Score** | **Judgement** | **Support for judgement** |  |  |  |
| **1-Description of the Active components included in the intervention** | 3 | More than one Component as a bundle | Education on condition, Follow-up reinforcement, exercise follow up, medication adherence support |  |  |  |
| **2-Description of Behaviour or actions of intervention recipients or participants to which the intervention is directed** | 3 | Multi-target | Medication adherence, communication with healthcare, excercise |  |  |  |
| **3-Description of Organisational levels and categories targeted by the intervention** | 1 | Single category | Patients |  |  |  |
| **4-The degree of tailoring intended or flexibility permitted across sites or individuals in applying or implementing the intervention** | 2 | Moderately tailored/flexible | Patient-specific education and emergency calls could be made |  |  |  |
| **5-Description of The level of skill required by those delivering the intervention in order to meet the intervention’s objectives** | 2 | Intermediate skills | Team received a training DVD that extended the content provided in written materials |  |  |  |
| **6-Description of the The level of skill required for the targeted behaviour when entering the included studies by those receiving the intervention, in order to meet the intervention’s objectives** | 1 | Basic skills | No specified skills require by participant |  |  |  |
| **Overall score** | 12 | **High complexity** |  |  |  |  |
| **Study authors:** Schmaderer *et al*. 2022, USA | | | |  |  |  |
| **Complexity** | **Score** | **Judgement** | **Support for judgement** |  |  |  |
| **1-Description of the Active components included in the intervention** | 3 | More than one Component as a bundle | Education on condition, Lifestyle education, Symptoms monitoring, Follow-up reinforcement |  |  |  |
| **2-Description of Behaviour or actions of intervention recipients or participants to which the intervention is directed** | 3 | Multi-target | Education on condition, Lifestyle education, Symptoms monitoring, Follow-up reinforcement |  |  |  |
| **3-Description of Organisational levels and categories targeted by the intervention** | 1 | Single category | Patients |  |  |  |
| **4-The degree of tailoring intended or flexibility permitted across sites or individuals in applying or implementing the intervention** | 3 | Highly tailored/flexible | mHealth app was quite inflexible while virtual visitis are moderately tailored/inflexible |  |  |  |
| **5-Description of The level of skill required by those delivering the intervention in order to meet the intervention’s objectives** | 2 | Intermediate skills | The principal investigator used a protocol guide for consistent training of all personnel. |  |  |  |
| **6-Description of the The level of skill required for the targeted behaviour when entering the included studies by those receiving the intervention, in order to meet the intervention’s objectives** | 1 | Basic skills | No specified skills require by participant |  |  |  |
| **Overall score** | 13 | **High complexity** |  |  |  |  |
| **Study authors:** Tulepbergenov *et al*., 2022, Kazakhstan | | | |  |  |  |
| **Complexity** | **Score** | **Judgement** | **Support for judgement** |  |  |  |
| **1-Description of the Active components included in the intervention** | 1 | One component | Medication adherence support |  |  |  |
| **2-Description of Behaviour or actions of intervention recipients or participants to which the intervention is directed** | 1 | Single target | Medication adherence |  |  |  |
| **3-Description of Organisational levels and categories targeted by the intervention** | 1 | Single category | Patients |  |  |  |
| **4-The degree of tailoring intended or flexibility permitted across sites or individuals in applying or implementing the intervention** | 2 | Moderately tailored/flexible | Mobile app |  |  |  |
| **5-Description of The level of skill required by those delivering the intervention in order to meet the intervention’s objectives** | 1 | Basic skills | No specified skills require by inventors |  |  |  |
| **6-Description of the The level of skill required for the targeted behaviour when entering the included studies by those receiving the intervention, in order to meet the intervention’s objectives** | 2 | Intermediate skills | Intervention group underwent a short training on how to use the application and the text instructions |  |  |  |
| **Overall score** | 7 | **Medium complexity** |  |  |  |  |
| **Study authors:** Välimäki *et al*.,2017, Finland | | | |  |  |  |
| **Complexity** | **Score** | **Judgement** | **Support for judgement** |  |  |  |
| **1-Description of the Active components included in the intervention** | 3 | More than one component as a bundle | Text reminder on medication adherence and self-care |  |  |  |
| **2-Description of Behaviour or actions of intervention recipients or participants to which the intervention is directed** | 2 | Dual-target | Medication adherence, Education on condition for selfcare |  |  |  |
| **3-Description of Organisational levels and categories targeted by the intervention** | 1 | Single category | Patients |  |  |  |
| **4-The degree of tailoring intended or flexibility permitted across sites or individuals in applying or implementing the intervention** | 2 | Moderatley tailored/flexible | Messages tailored upon patient needs |  |  |  |
| **5-Description of The level of skill required by those delivering the intervention in order to meet the intervention’s objectives** | 2 | Intermediate skills | Research nurses had 2 days training and 2 days of training updates during the intervention |  |  |  |
| **6-Description of the The level of skill required for the targeted behaviour when entering the included studies by those receiving the intervention, in order to meet the intervention’s objectives** | 1 | Basic skills | No specified skills require by participant |  |  |  |
| **Overall score** | 11 | **Medium complexity** |  |  |  |  |
| **Study authors:** Widmer *et al.,* 2017, USA |  |  |  |  |  |  |
| **Complexity** | **Score** | **Judgement** | **Support for judgement** |  |  |  |
| **1-Description of the Active components included in the intervention** | 3 | More than one Component as a bundle | Lifestyle education, Vital sign monitoring, Blood test monitoring, medication adherence support |  |  |  |
| **2-Description of Behaviour or actions of intervention recipients or participants to which the intervention is directed** | 2 | Dual-target | Medication adherence, Education on condition for selfcare |  |  |  |
| **3-Description of Organisational levels and categories targeted by the intervention** | 1 | Single category | Patients |  |  |  |
| **4-The degree of tailoring intended or flexibility permitted across sites or individuals in applying or implementing the intervention** | 2 | Moderatley tailored/flexible | Prompted messages upon patient abnormalities |  |  |  |
| **5-Description of The level of skill required by those delivering the intervention in order to meet the intervention’s objectives** | 2 | Intermediate skills | Team Trained on using the DHI web portal |  |  |  |
| **6-Description of the The level of skill required for the targeted behaviour when entering the included studies by those receiving the intervention, in order to meet the intervention’s objectives** | 1 | Basic skills | No specified skills require by participant |  |  |  |
| **Overall score** | 11 | **Medium complexity** |  |  |  |  |
| **Study authors:** Zhang et al., 2019, China |  |  |  |  |  |  |
| **Complexity** | **Score** | **Judgement** | **Support for judgement** |  |  |  |
| **1-Description of the Active components included in the intervention** | 3 | More than one Component as a bundle | Medication adherence, education on condition, medication reconciliation, adverse reactions report, pain management |  |  |  |
| **2-Description of Behaviour or actions of intervention recipients or participants to which the intervention is directed** | 2 | Dual-target | Medication adherence, pain management |  |  |  |
| **3-Description of Organisational levels and categories targeted by the intervention** | 1 | Single category | Patients |  |  |  |
| **4-The degree of tailoring intended or flexibility permitted across sites or individuals in applying or implementing the intervention** | 2 | Moderatley tailored/flexible | Prompted messages upon patient abnormalities |  |  |  |
| **5-Description of The level of skill required by those delivering the intervention in order to meet the intervention’s objectives** | 1 | Basic skills | No specified skills require by health care team |  |  |  |
| **6-Description of the The level of skill required for the targeted behaviour when entering the included studies by those receiving the intervention, in order to meet the intervention’s objectives** | 1 | Basic skills | 10 minutes training |  |  |  |
| **Overall score** | 10 | **Medium complexity** |  |  |  |  |
